# Parental Stress Provoked by Short-Term School Closures During the Second COVID-19 Lockdown

**DOI:** 10.1177/0192513X211041987

**Published:** 2023-01

**Authors:** Isabelle May, Sarah Awad, Matthias S. May, Albert Ziegler

**Affiliations:** 1 Department of Educational Psychology and Research on Excellence, 9171 Friedrich-Alexander-Universität Erlangen-Nürnberg (FAU), Nuremberg, Germany; 2 Medical Faculty, 9171 Friedrich-Alexander-Universität Erlangen-Nürnberg (FAU), Erlangen, Germany

**Keywords:** COVID-19, childhood, family relations, parental stress, exposed children

## Abstract

Governments of numerous countries implemented school closures to contain the COVID-19 pandemic. Several investigations have shown the negative impact of social-distancing policies and school closures on children worldwide. Recently, research also demonstrated adverse effects on adults’ well-being. The development of children is strongly affected by their parent’s emotional state. The present study aimed to examine parental stress levels caused by a short period of homeschooling in December 2020 in Germany. A structured survey was set up and distributed randomly via social media and parent associations. We observed a significant increase in stress and concerns. Family conflicts significantly increased, social isolation was feared, and powerlessness and helplessness ascended. Risk factors were parental education levels, parental working time, and teaching features like the frequency of feedback, correction, and accessibility.

## Introduction

Approximately three billion people have been affected by containment policies due to the COVID-19 pandemic since the beginning of 2020. These measures have significantly reduced infection rates and have saved millions of lives (Hsiang et al., 2020). The extent of the social consequences caused by these policies, such as school closures and exit restrictions, on the human psyche is not yet clear. Studies showed an overall reduced mental well-being among the general population in Germany already in May 2020. Higher levels of anxiety, stress, and depression than before the COVID-19 pandemic were reported (Fontanesi et al., 2020; Haas, 2020; Horesh & Brown, 2020; Zacher & Rudolph, 2020). Several studies on effects on children have demonstrated substantial negative consequences on their psychological well-being (Fegert, Vitiello, Plener, & Clemens, 2020; Haas, 2020; Ravens-Sieberer et al., 2020).

### Distance Learning in Germany

Due to the school closings that began in March 2020, periodical distance teaching has partially replaced face-to-face teaching in Germany. The parents of approximately three million elementary-school pupils became “auxiliary teachers” overnight (Müller, Samtleben, Schmieder, & Wrohlich, 2020). This situation caused a redefinition of the parental role in the teaching and learning process (Voss & Wittwer, 2020). In addition to providing technical support for video conferences and using a wide variety of learning media, parents scheduled class days, printed out worksheets, explained exercises, and corrected homework. For most parents, coordination of these new tasks with work and household responsibilities was perceived as an additional burden (Bujard, Laß, Diabaté, Sulak, & Schneider, 2020). Contact restrictions, health, financial worries, and new family situations caused decreased well-being and a greater extent of arguments in some families (Vodafone Stiftung, 2020). In the “Corona and Psyche” study on children and adolescents’ mental health and quality of life during the COVID-19 pandemic, 65% of the surveyed children and adolescents stated that school was more strenuous than before.

Moreover, 37% of parents reported frequently escalating arguments with their children (Ravens-Sieberer et al., 2020). One-quarter of parents reported a more complicated relationship with their children due to distance learning in a nationwide parent survey in Germany (Wildemann & Hosenfeld, 2020). Furthermore, recent findings indicate a substantial influence of emotions on learning success in distance learning (Huber & Helm, 2020). The actions proposed by the World Health Organization and other experts to reduce these adverse effects include maintaining habitual processes within the family, the continuity of educational measures, and a positive family climate (Haas, 2020).

### Parental Stress

According to Lazarus, psychological stress results in one’s relationship with the environment, which the person assesses concerning their well-being and may result in coping options that overwhelm or strain the person (Lazarus & Folkman, 1984). Various factors can determine parental stress via situation assessments, assess available coping strategies, and reassess the situation after adaptation. Subjective experiences of stress emerge when coping resources are insufficient (Tischler & Petermann, 2011).

**Figure 1.**
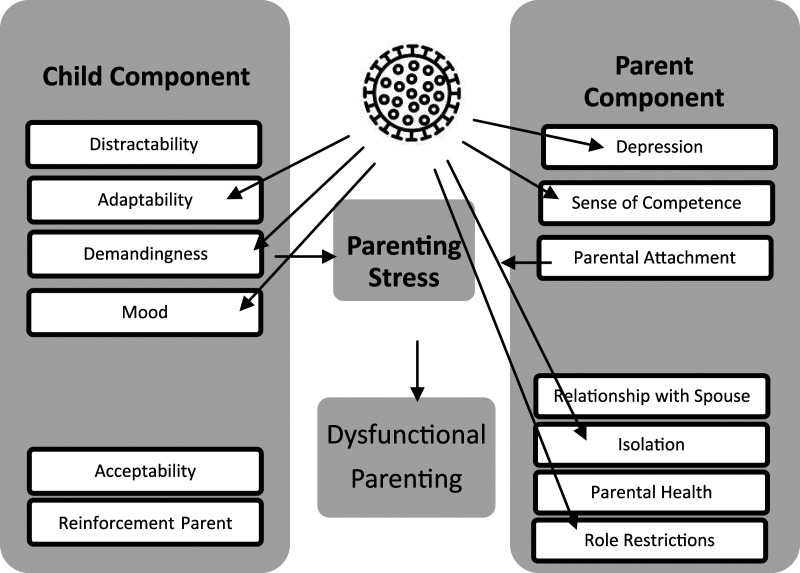
COVID-19–distance learning–stress model adapted from the parenting stress model in Tröster, 2011, modified from Abidin [1995, *p*. 30].

Most academic articles refer to parental stress due to the divergence between the current requirements placed on parents concerning their parenting role and the perceived means at their disposal to meet those demands. If parents feel stressed or burdened by their perceived expectations regarding responsibilities in education and the upbringing and care of their child due to immediate difficulties, a unique form of stress arises: parental stress or parental strain (Tröster, 2011). About school closings and distance teaching during the COVID-19 pandemic, we have developed the following parenting–distance learning–stress model based on Abidin’s modified parenting stress model (Figure 1).

Adaptability describes the child’s ability to adapt to changes in the environment (Tröster, 2011). The transformation of schooling due to school closures has uniquely affected children’s adaptability. In particular, avoidance behavior, which may indicate a lack of adaptability, may present a challenge for parents (Tröster, 2011). Demandingness indicates a child’s direct demand on parents due to developmental delays, illness, or personality (Tröster, 2011). Without the daily professional support of teachers in education, these children’s parents are stressed in two ways. A child’s negative mood can also be a source of parental stress (Tröster, 2011). School closures may affect children’s moods in different ways. It is difficult for parents with depression to mobilize the energy necessary to react appropriately to their child’s needs (Tröster, 2011). Scientific investigations have shown that social distancing may induce depression among risk groups (Haas, 2020). Doubts in one’s competence regarding education may also provoke parental stress (Tröster, 2011). The unique situation of school closings and parental support in learning has generated a new parental role in the learning process. To successfully manage this new homeschooling situation, parents need various skills. A lack of social support also leads to parental stress (Tröster, 2011). Contact restrictions have led to a substantial reduction in families' natural social support, for example, grandparents and neighbors.

Perceived role restrictions induce parental strain (Tröster, 2011). The unique lockdown situation has increased parental role restrictions. Distance learning can also evoke positive emotions in that the new task is seen as a learning aid and as a challenge, and good management of this task leads to positive feelings. Because the conditions for all families have changed in some areas, parents’ perceptions of stress should not vary significantly. In contrast, one could also suspect that the experience of stress in certain groups has changed.

### Aim of This Study

The government announced another nationwide lockdown in Germany on December 12, 2020. Therefore, all German schools had to be closed from December 15, 2020, until further notice. We expected a higher stress level among parents during the short-term school closures between December 16, 2020, and December 22, 2020. This period, clearly delimited by the Christmas holidays, was chosen to assess the effects of short-term school closure on parental stress and teaching changes since the lockdown in spring 2020. We expected that two controversial results might have influenced the homeschooling situation: adaptations, improvements, and lessons learned from the first lockdown on one side and a short preparation time of only 3 days on the other side. The aim was to evaluate possible influencing factors on the parental experience of stress for this unique situation. We assume that modifications of the teaching methods and improvements of students’ and teachers’ technical equipment, based on the experiences from closures in spring 2020, positively impacted families’ stress perception. At best, these results should have helped to improve the family atmosphere and generate disruption-free learning for children and emotional relief. The following research hypotheses arose:

h0. Short-term school closures do not cause parental stress. (Stress)
h1. Working situations influence parental stress levels. (Working situation)
h2. Problems regarding time constraints increase stress. (Time)
h3. Different teaching features influence parental stress. (Teaching)
h4. Loss of social support increases parental stress. (Support)
h5. Children’s competence influences parental stress. (Children)

## Data and Methods

### Participants

An online survey (www.umfrageonline.com, enuvo GmbH, Zürich, Switzerland) was randomly distributed to primary school parents in Germany via social networks over 10 days (December 21–31, 2020) after the sudden-onset of school closures for the last week before the Christmas holidays (December 16–22, 2020). Participation was voluntary. A total of 521 started the survey, and 469 parents (90%) completed the survey. We included participants from a single administrative region (Bavaria) to limit the potential bias from different federalist situations or school systems finally resulting in 313 data sets for the statistical analysis, excluding all other participants. Table 1 presents demographic data for the families participating in the study. The sample consisted mainly of mothers (83%), part-time workers (64%), and parents with two children (59%).

**Table 1.** Descriptive Data.

		*n*	%
Gender	Male	51	16
	Female	261	83
Age (years)	18–30	3	1
	31–40	159	51
	41–50	141	45
	>51	12	4
Education	No education	2	1
	Secondary school	50	16
	Associated degree	90	29
	Bachelor’s degree	58	18
	Master’s degree/doctoral degree	115	37
Working hours	Not working	39	12
	Part-time up to 50%	77	24
	50% or more	125	40
	Full time	74	24
Work situation	100% home office	117	37
	Switching from home office to in-person	102	32
	100% in-person	96	31
Number of children	1	72	23
	2	185	59
	3	50	16
	4	5	2
	5	3	1
Family situation	Single parent	22	7
	Divorced in an alternate model	9	3
	Two-parent family	284	90
Support in hours per day	<1	34	11
	2	80	26
	3	96	31
	4	61	20
	5	29	9
	>5	12	4

### Instrument

The survey contained 31 questions about demographic data (*n* = 9) and about the family situation during distance learning (*n* = 22, see Supplementary Appendix A). The latter was structured into stress items (*n* = 3), teaching features (*n* = 8), working situation (*n* = 2), time (*n* = 2), childrens’ competencies (*n* = 4), and support (*n* = 3). It was available in English and German language.

#### h0: Stress

Three questions depicted stress appraisals. First, coping with stress related to distance learning was assessed by four items (Q 20: motivation, scheduling, technology, and explanation). The Cronbach’s alpha value of these four items was α = 0.83, signalizing good internal consistency. Second, we evaluated parental concerns by three items (Q 21: evolvement of knowledge gaps, loss of social interactions, and loss of motivation). The internal consistency for parental concerns was acceptable, α = 0.78. Third, stress manifestation was measured with three items (Q 22: isolation, competence, role restriction) referring to the German version of the Parenting Stress Index (PSI, Tröster, 2011). The Cronbach’s alpha consistency level was also good (α = 0.85). All characteristics were evaluated on a four-point Likert scale (1 = strongly disagree, 2 = disagree, 3 = agree, 4 = strongly agree).

#### h1: Working Situations

The working situation of the parents may be a potential influencing factor on the perceived stress level. Parents were asked about their current working place (Q1, 1 = 100% home-office, 2 = alternating home-office and presence, 3 = 100% presence) and working hours (Q2, 1 = not working, 2 = part-time up to 50%, 3 = 50% or more, 4 = full time).

The education level from the survey’s demographic part (Q29) was also included for the post-hoc analysis of this hypothesis.

#### h2: Time

Distance learning support competes with personal spare time and professional and domestic work. Additional time expenses per day were assessed in six categories (Q5; <1 h, 1–2 h, 2–3 h, 3–4 h, 4–5 h, >5 h). The perceived difficulty level to make this time available during regular working days was evaluated on the same four-point Likert scale as mentioned above (Q6).

#### h3: Teaching

Technological facilities may be a vital issue to maintain the communication between the teachers, the pupils, and the parents. According to the communication recipient, teaching features were categorized: children (Q8) or parents (Q9). We assessed the way of communication separately as mail (Q8/9a), phone (Q8/9b), and video-chat (Q8/9c). Frequency of correction (Q10), the supply of a weekly schedule (Q11), and teachers’ accessibility (Q13) were used to indicate features of education, measured on a 4-point Likert scale (1 = never, 2 = rarely, 3 = occasionally, and 4 = regularly). The parents specified their support level before school closings (Q19) and during distance learning (Q15) for four items: motivation, scheduling, technology, and explanation. We also asked the parents about expected school support measures (Figure 1) to ease their work with the children and reduce stress (Q23, Q24).

#### h4: Support

External support (Q16) has a high potential to relieve family stress. Items about motivation, scheduling, technology, and explanation indicated the regular support of the parents from outside on the four-point Likert scale. A good consistency (α = 0.86) was calculated for the support items. The ability to ask for help is a sign of resilience. Parental ideas about where they can get help were asked (Q14) on a dichotomous scale.

#### h5: Children

Family interactions and competencies may be influential as well. The frequency of arguments about distance learning was assessed on the four-point Likert scale (Q17). Before the closings (Q18), the situation was evaluated by four items (Q18a: desire to go to school, Q18b: learning and Q18c: scheduling competencies, and Q18d: frequency of domestic arguments) measured on a four-point Likert scale. It was expected that children with a higher competence in self-regulated learning cause less strain on the family. The Cronbach’s alpha for child competence items was acceptable (α = 0.76). Another question focused on how much the children long for their classmates and teachers (Q12).

## Statistics

The statistical evaluation was carried out using the “psych” package in R version 4.0.3 (R Core Team, 2020). Non-parametric *X*^2^ tests were performed to evaluate the distribution of ordinal-scaled responses. The distribution of most items was not normal. Descriptive statistics are provided as median, mean, and *SD*. Cronbach’s alpha was calculated for items with multiple questions to test for internal consistency. Kruskal–Wallis tests were used to evaluate differences between the questions from h0 about stress related to distance learning (Q20), parental concerns (Q21), and stress manifestation (Q22) and to compare the parental level of education in h1 (Q29). Wilcoxon rank-sum tests were used to evaluate the impact of all other potential stressors (h1-6) to compare a “non-stressed” (Likert 1/2) subgroup with a “stressed” (Likert 3/4) subgroup from h0, to evaluate the impact of all other potential stressors (h1–h6). Wilcoxon rank-sum tests were also used to compare the parental level of teaching support before school closures and during distance learning. Descriptive statistics are provided as percentages. The level of significance is 5%. Due to the limited space, these latter non-significant results are not presented in detail.

## Results

### h0: Stress

**Figure 2.**
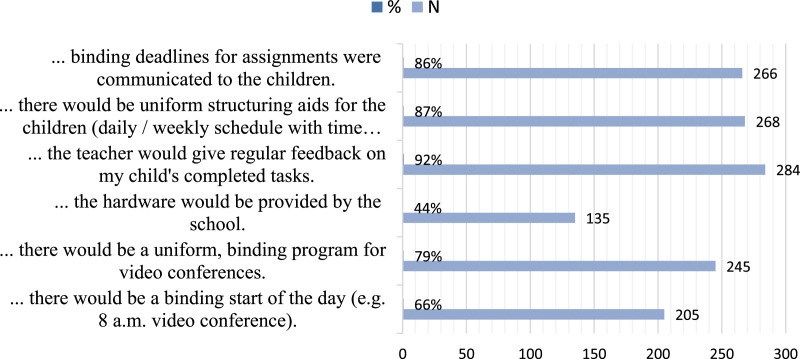
Parental demands for school support (Q23, all respondents).

Table 2 and Figure 2 provide detailed results for h0 questions (*Short-term school closures do not cause parental stress*).

**Table 2.** Data Report of all Stress Items.

Items	Questions	Mdn	*M*	*SD*	*X*^2^	*p*
Stress related to distance learning	Q20a: motivation	3	3.05	0.98	97.56	<0.001
	Q20b: scheduling	3	2.57	1.05	10.40	<0.001
	Q20c: technology	3	2.50	1.08	1.16	0.015
	Q20d: explanation	3	2.38	1.10	4.02	0.76
Parental concerns	Q21a: evolvement of knowledge gaps	3	2.95	1.05	61.09	<0.001
	Q21b: loss of social interactions	3	3.00	0.99	65.33	<0.001
	Q21c: loss of motivation	3	3.20	0.94	128.70	<0.001
Stress manifestation (PSI)	Q22a: isolation	3	3.06	0.92	87.65	<0.001
	Q22b: competence	3	3.28	0.92	165.67	<0.001
	Q22c: role restriction	3	3.14	0.99	112.57	<0.001

*N* = 313; df (3).

The average stress level over all items was relatively high (*Mdn* = 3, *M* = 2.64, *SD* = 0.88). Motivation (20a) had the highest mean value (*M* = 3.05, *SD* = 0.98), which was significantly higher than the perceived stress from the other items 20b–c (*p* < 0.001). Significantly, more parents (75%) felt stressed by motivating their children to work (Q20a, Likert 3 or 4, *p* < 0.001). Only 25% reported low or no stress from this motivational task (Likert 2 or 1). More than half of the parents (62%) felt strained from scheduling the homeschooling day (Q20b). This trend was also statistically significant, *p* < 0.001. The distributions of stress manifestation from technical problems (Q20c) and professional explanation (Q20d) were non-significant.

The average level of concerns (*Mdn* = 3, *M* = 3.05, *SD* = 0.83) was even higher than the perceived stress rating from Q20. The loss of children’s motivation displayed the highest mean values (21c, *Mdn* = 3, *M* = 3.20, *SD* = 0.94), significantly higher than the other items, *p* < 0.001. About 64% of all parents had concerns about the evolvement of knowledge gaps (Q21a), *p* < 0.001*.* More than two-thirds (69%) of the parents reported concerns about the loss of social skills (Q21b, *p* < 0.001), and 67% of the parents had sorrows about motivational deprivation (Q21c, *p* < 0.001).

The average stress level of PSI-adapted items was very high (*Mdn* = 3, *M* = 3.16; *SD* = 0.81) and highest among h0 questions. Doubts on the own competence (Q22b) had the highest mean (Mdn = 3, *M* = 3.28, *SD* = 0.92). Three quarters of parents (75%) agreed with the isolation statement (Q22a, Figure 2, *p* < 0.001) and felt stress from role restrictions (Q22c, *p* < 0.001). Even more had no trust in their own competences (81%, Q22b, *p < 0.001*)*.*

### h1: Working Situations

Working situations were homogeneously distributed, *X*^2^ = 2.38, *p* = 0.30. (Table 1). Stressed parents’ working places were comparable to non-stressed parents working places*.* Most parents worked part-time (64%), and only a minority were not working (12%). The distribution of working hours in all stress-related items (h0) was comparable (all *p* > 0.05). More than half of our collective had a university degree (55%). There was a statistically significant link between the parental level of education (Q29) and concerns about the evolvement of knowledge gaps (Q20a), *p* < 0.001. Higher education comes along with more concerns. All other stress-related items were independent of the parental level of education (all *p* > 0.05).

### h2: Time

Half of all parents spent 3 hours per day supporting their children in school issues (Q5, *M* = 3.0, *SD* = 1.26, *X*^2^ = 113, *p* < 0.001). Parents who had problems coping with stress related to distance learning (Q20), parents with concerns (Q21), and parents with high PSI manifestations (Q22) spent more time supporting distance learning than non-stressed parents, *p* < 0.001 (Table 3).

**Table 3.** Data Report of Hypothesis h3–h6. Results Wilcoxon Test for all Stress Items (Stressed Parents vs. Non-Stressed Parents).

H	Median	Question	Stress items	Non-Stressed (M)	Stressed (M)	Sum of ranks	*p*
h3: Time	3	Q5: amount of time	Q 20	2.72	3.21	14410	<0.001
			Q 21	2.62	3.14	11304	0.0014
			Q 22	2.67	3.1	9584.5	0.01
	3	Q6: time finding problem	Q 20	2.24	3.23	17066	<0.001
			Q 21	2.2	3.15	14000	<0.001
			Q 22	2.01	3.14	12754	<0.001
h4: Teaching	1	Q8: communication: child–teacher Q8a: via mail	Q 20	1.78	1.71	11824	0.985
			Q 21	1.77	1.72	9235	0.911
			Q 22	1.97	1.68	6814.5	0.047
	1	Q8b: via video chat	Q 20	1.98	1.81	10700	0.123
			Q 21	2.18	1.78	7528.5	0.009
			Q 22	2.17	1.8	6802	0.049
	1	Q8c: via phone	Q 20	1.43	1.35	11757	0.931
			Q 21	1.29	1.41	9959	0.143
			Q 22	1.42	1.37	7692.5	0.586
	2	Q9: communication: parent–teacher Q9a: via mail	Q 20	2.34	2.46	11842	0.967
			Q 21	2.34	2.46	9790.5	0.344
			Q 22	2.29	2.47	8824	0.165
	3	Q9b: via video chat	Q 20	3.65	3.59	11560	0.667
			Q 21	3.55	3.63	9576	0.425
			Q 22	3.55	3.63	8290	0.503
	3	Q9c: via phone	Q 20	3.35	3.3	11202	0.394
			Q 21	3.23	3.35	9722	0.377
			Q 22	3.28	3.33	8283.5	0.591
h4: Teaching	3	Q10: correction	Q 20	2.58	2.48	11252	0.46
			Q 21	2.82	2.42	7516.5	0.01
			Q 22	2.92	2.42	6156.5	0.004
	4	Q11: weekly schedule	Q 20	3.83	3.75	11201	0.20
			Q 21	3.83	3.77	8846.5	0.44
			Q 22	3.78	3.78	7976.5	0.98
	3	Q13: teachers’ accessibility	Q 20	3.24	2.93	9341	<0.001
			Q 21	3.38	2.94	6367.5	<0.001
			Q 22	3.39	2.97	5751.5	<0.001
	3	Q15: parental support Q15a: motivation	Q 20	3.31	3.81	15248	<0.001
			Q 21	3.27	3.71	11958	<0.001
			Q 22	3.31	3.67	9831	<0.001
	3	Q15b: scheduling	Q 20	2.95	3.38	16086	<0.001
			Q 21	2.98	3.44	11942	<0.001
			Q 22	2.94	3.43	10324	<0.001
	3	Q15c: technology	Q 20	2.99	3.57	15676	<0.001
			Q 21	3.01	3.45	11716	<0.001
			Q 22	3.04	3.41	10014	<0.001
	3	Q15d: explanation	Q 20	3.16	3.64	15412	<0.001
			Q 21	3.14	3.55	11675	<0.001
			Q 22	2.56	2.71	10426	<0.001
	3	Q19: parental support before Q19a: motivation	Q 20	2.51	2.71	13136	0.077
			Q 21	2.61	2.63	9238.5	0.912
			Q 22	2.58	2.65	8266	0.629
	2	Q19b: scheduling	Q 20	2.03	2.31	13495	0.025
			Q 21	2.06	2.24	9989.5	0.214
			Q 22	2.09	2.22	8398.5	0.487
	3	Q19d: explanation	Q 20	2.54	2.67	12744	0.21
			Q 21	2.6	2.62	9329	0.803
			Q 22	2.57	2.63	8240.5	0.656
h5: Support	1	Q:14 obtain for assistance	Q 20	1.31	1.18	13266	0.017
			Q 21	1.31	1.1	11116	0.002
			Q 22	1.3	1.13	9338	0.006
	3	Q16a: motivation	Q 20	2.54	2.77	13086	0.9265
			Q 21	2.37	2.78	11164	0.002
			Q 22	2.71	2.56	8551	0.34
	3	Q16b: scheduling	Q 20	2.05	2.79	14223	0.001
			Q 21	2.64	2.64	11024	0.005
			Q 22	2.58	2.37	8788	0.18
	3	Q16c: technology	Q 20	2.05	2.65	15152	<0.001
			Q 21	2.01	2.54	11434	<0.001
			Q 22	2.28	3.1	17447	<0.001
	3	Q16d: explanation	Q 20	2.42	2.77	14044	0.003
			Q 21	2.26	2.75	11624	0.002
			Q 22	2.55	2.66	8742	0.21
h6: Children	3	Q17: arguments	Q 20	2.28	3.12	17447	<0.001
			Q 21	2.24	2.95	12868	<0.001
			Q 22	1.98	2.98	12406	<0.001
	3	Q18a: like to go to school	Q 20	3.66	3.64	11976	0.78
			Q 21	3.76	3.61	8345	0.13
			Q 22	3.7	3.63	7405	0.26
	3	Q18b,d: self-regulation	Q 20	2.92	2.75	10798	0.16
			Q 21	2.85	2.8	8957	0.74
			Q 22	2.98	2.77	7106	0.15
	2	Q18c: arguments before closures	Q 20	1.79	2.02	13520	0.01
			Q 21	1.96	1.83	10098	0.14
			Q 22	1.9	1.93	8180	0.72
	3	Q12: lack of communication	Q 20	2.93	3.24	9341	<0.001
			Q 21	2.95	3.38	6367	<0.001
			Q 22	2.97	3.39	5751	<0.001

*N* = 313; df (3).

Most parents (70%) reported problems to find time to support their children (Q6, *M* = 2.9, *SD* = 0.93, *X*^2^ = 65, *p* < 0.001). Time management is a substantially bigger problem for the stressed group in all stress-related questions, as shown in Table 3.

**Figure 3.**
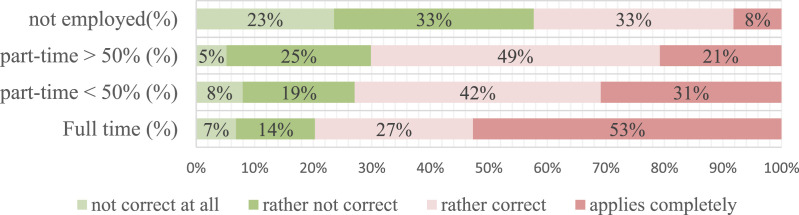
Parental problems in finding time (Q6, all respondents).

Interestingly, parents with higher working times had significantly more problems providing distance learning support time; *p* < 0.001 (Figure 3). The highest values were found for full-time employees (80%). Most part-time working parents were also affected (70%), but more than half of the unemployed parents reported no or low-stress levels (60%).

### h3: Teaching

Detailed stress-related statisitcs are provides in Table 3. More than half of all parents reported that their children had no contact with their teachers (Likert 1) via mail (56%, Q8a: *M* = 1.74, *SD* = 0.95, *p* < 0.001), via video chat (53%, Q8b: *M* = 1.87, *SD* = 1.08, *p* < 0.001) or via phone (72%, Q8c: *M* = 1.38, *SD* = 0.70, *p* < 0.001). Parental teacher contacts were more frequent. More than two-thirds of all parents communicated with their teachers regularly (Likert 4) via mail (72%, Q9a, *M* = 3.16, *SD* = 0.78, *p* < 0.001). Phone calls were also frequently used (51%, Q9b, *M* = 3.19, *SD* = 0.82, *p* < 0.001), but video chats were rather rare (14%, Q9c, *M* = 2.43, *SD* = 0.93, *p* < 0.001). There was no significant relation between parental stress levels and ways of communication.

Parents reported that 29% of teachers never (Likert 1) corrected pupil’s exercises, but in 54% corrections were provided occasionally or regularly (Q10, *M* = 2.52, *SD* = 1.26, *p* < 0.001). Exercises from less concerned (Q21) and less stressed (Q22) parents were corrected more frequently than the more concerned and stressed parents.

Most of all families (86%) received a weekly schedule from their teachers (Q11, *M* = 3.78, *SD* = 0.59, *p* < 0.001). The frequency of weekly schedules was comparable in the stressed and non-stressed subgroups.

Overall, 75% of all families reported a good teachers’ accessibility (Q13, *M* = 3.05, *SD* = 0.84, *p* < 0.001). Stressed parents’ subgroups from all questions reported significantly lower teachers’ accessibility.

Approximately 90% of all parents were motivating their child to learn continuously (Q15a, *M* = 3.60, *SD* = 0.75). More stressed parents in all three stress items were motivating their children more often than non-stressed parents.

There was a significant difference in parental support in motivating the child to learn before (Q19a, *M* = 2.63) and after (Q15a, *M* = 3.60) school closures, *p* < 0.001. Also, the increase in required scheduling support (Q19b and Q15b, *M* = 2.19 vs. 3.33, *p* < 0.001) and learning explanation (Q19d and Q15d, *M* = 2.62 vs. 3.45, *p* < 0.001) was significant. Parental technical support during distance learning was quite high (82%, Q15c, *M* = 3.33, *SD* = 0.9, *p* < 0.001).

**Figure 4.**
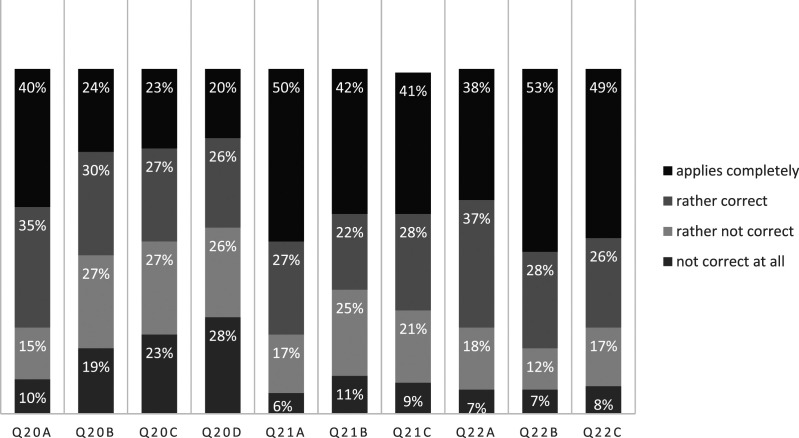
Stress items (Q20, Q21, Q22 all respondents).

Almost all parents suggested regular feedback, binding submission dates for exercises, and structuring aid to improve distance learning (Q23). Still, two-thirds considered a uniform, binding program for videoconferences as desirable support to reduce stress. More than half of all parents aimed for a binding start every school day (Figure 4).

### h4: Support

We did not find any relevant differences in the frequency of responses (all *X*^2^ > 0.05). Interestingly, parents in several stress-related subgroups received significantly more external support than in the non-stressed subgroups. In total, 72% of all parents knew where to obtain assistance (Q14, *M* = 1.26, *SD* = 0.45). Stressed parents of all three item subgroups had significantly fewer ideas to find assistance (Q14, Table 3).

### h5: Children

More than two-thirds of the parents (72%) reported that their children liked to go to school before the closings (Q18a, *M* = 3.65, *SD* = 0.65, *p* < 0.001).

About 82% of all parents described that their children were used to learn independently before school closures (Q18b, *M* = 3.22, *SD* = 0.80, *p* < 0.001), and only 22% of families used to argue about school issues (Q18c, *M* = 1.93, *SD* = 0.83, *p* < 0.001). Most of the children (70%) were able to schedule their homework in the afternoon independently (Q18d, *M* = 2.82, *SD* = 0.86, *p* < 0.001).

Around 60% of all parents reported disputes with their children in distance learning situations (Q17, *M* = 2.77, *SD* = 0.96, *p* < 0.001). Stressed parents’ subgroups from all stress questions reported significantly higher quarrel rates with their children due to distance learning. There was a significant increase compared to the dispute frequency before distance learning (Q18c, *M* = 1.93, *p* < 0.001).

Most of all parents (87%) stated that their children miss their teachers and classmates (Q12, *M* = 3.46, *SD* = 0.79, *p* < 0.001). Perceived lack of children’s social interactions was also significantly higher in stressed parents (Table 3).

## Discussion

Parental stress caused by short-term school closures increased. Problems regarding time constraints are an explanation for these results. Finally, distance learning requires intensified support for the pupils. Parents cover an average of 3 hours per day to compensate for the significantly increased need for motivation, scheduling, and professional advice, complemented by technical help. The exact time amount was previously reported in the Vodafone study during the spring school closures in Germany in 2020. However, at that moment, only less than half of all parents reported that time management was a substantial problem for them (Vodafone Stiftung, 2020). This is contradictive with the present outcomes, which indicate a high stress level caused by time-finding problems. These different perceptions of strain may result from the date of the survey. As the Vodafone survey was carried out 3 weeks after school closures in April 2020, parents compensated time requirements by holidays and reduction of overtime hours. At the end of 2020, a year with broad cumulative school closures, most working parents reached their time management limit. Interestingly, in spring 2020, almost three-quarters of the parents were aware of difficulties maintaining their learning support in extended distance learning throughout the year (Vodafone Stiftung, 2020). The present study confirms this prediction.

Another explanation for increased parental stress might be different teaching features caused by school closures. One is the changed communication. So due to distance learning, family interactions increased. Inversely, the communication between pupils and teachers was scarce and switched to more interaction between parents and teachers. The parental role underwent a severe change during distance learning. Hence, we found a remarkably high average stress perception among primary school children’s parents during the short-term school closures in December 2020. This supports the thesis of Voss and Wittwer, who already discussed the increasing role of parental support and time requirements after the first distance learning period to potentially provoke increased parental stress (Voss & Wittwer, 2020).

Furthermore, different parental working situations led to higher stress levels during school closures. Several statistical surveys have shown that certain parent groups are more susceptible to this stress due to personal specifications. So, single parents, families with little education, families receiving social transfers, families with a migration background, and parents with jobs of systemic importance were previously identified as high-risk groups (Bujard et al., 2020; Geis-Thöne, 2020; van Ackeren, Endber, & Locker-Grütjen, 2020). In contrast, Porsch and Porsch (2020) determined a slight tendency among women, parents with several schoolchildren, working people, and parents with a higher educational qualification tend to be more stressed. Our study does not replicate all of these items. However, we found that working people, regardless of the working hours and parents with higher educational qualifications, felt more burdened.

In addition, consequences of containment policies led to a higher stress perception. So parents who felt more stressed stated that their children felt strained by the consequences of social restrictions (Langmeyer, Guglhör-Rudan, Naab, Urlen, & Winklhofer, 2020). This statement has been made more precise in the present study. Stressed parents reported that their children missed their classmates and teachers. At the same time, the parents mentioned concerns about their children’s social skills. Parents are strongly concerned about the consequences of children’s lack of regular scholarly social contacts.

Furthermore, we identified motivational problems and scheduling as potential risk factors that cause parental stress. Parental support in motivation and scheduling increased significantly compared to the times before school closures. More stressed parents helped their children more to motivate them. A lack of children’s motivation to learn and work led to parental stress. Voss and Wittwer also discussed the critical role of motivation for effective distance learning after the first lockdown (Voss & Wittwer, 2020). As motivation poses a source of stress for families, we demand some school system solutions. One could be an increase in the communication frequency between teachers and children, for example, via phone or video chat. It is noteworthy that the parents had very uniform ideas about helpful support from schools, such as the desire for a daily start together and continuous feedback. Porsch and Porsch (2020) also identified parental demands for adequate feedback during the first school closures in spring 2020.

The German “Schulbarometer” discovered that teaching features as feedback and communication influenced distance learning while school closures in spring 2020. Immediate and individual feedback led to higher positive emotions while distance learning and more learning time (Huber et al., 2020). Parents perceiving well-realized feedback reported high-quality distance teaching (Huber et al., 2020) and less strain (Porsch & Porsch, 2020). Feedback is one of the most potent influences on school achievements; d = .72 (Hattie, 2008). The present study underlines the importance of a sophisticated feedback strategy for distance learning. Well-implemented feedback may improve scholastic performance and contribute to a better family atmosphere.

The educational policy measures such as investments in digitalization, provision of teacher training measures, technical equipment for pupils and teachers, as well as didactic and methodological requirements for a procedure in the event of further school closings could not yet have any effect on a reduced experience of stress within families in the short period of this study.

### Limitations

We must consider limitations for interpreting our results: First, the questionnaire’s distribution was random, and the number of participants was low. This sample is not representative.Second,stressed parents certainly had a stronger motivation to complete this questionnaire. Extremely stressed parents could have problems finding time to fill out the survey. As the survey was distributed via the internet, families without any digital terminal could not take part. Although we offered an English version, it did not cover the linguistic diversity of immigrants living in Germany. Third, the natural pre-Christmas stress may bias the respondents’ perception of stress. Fourth, the children’s feelings, worries, and needs were not assessed directly but only through the parents’ impressions. Fifth, the concerns related to health threats, financial hardships, and interpersonal disorders were not explicitly asked. Sixth, the teachers were also surprised by the closures and could not apply their whole didactical repertoire very spontaneously. Last, there is no generalizability to other countries or educational settings.

## Conclusion

School closures caused a higher parental stress level. The major challenge was finding sufficient time to support distance learning. Preventive items seem to be extra available time for the parents, external help, and communication with the teachers. Parents hope for regular feedback from the teachers and more binding sequences and exercises to relieve domestic stress. The implementation of political measures as reinforcement of employee rights would relieve this situation. Educational interventions to motivate pupils like stimulating learning situations, fast feedback, and strict inspection are now necessary. Video conferences in regular intervals encourage interaction and the social community.

Moreover, teachers’ different verbal advice help schedule the homeschooling day and other children’s digital presence motivate them to work and learn. Teaching features influence parental strain. A practical application of feedback, communication, and scheduling tools while distance learning may reduce parental stress and improve the family atmosphere. Furthermore, it enhances a harmonic family climate the child’s ability to learn.

## Supplemental Material

sj-pdf-1-jfi-10.1177_0192513X211041987 – Supplemental Material for Parental Stress Provoked by Short-Term School Closures During the Second COVID-19 Lockdown Click here for additional data file. Supplemental Material, sj-pdf-1-jfi-10.1177_0192513X211041987 for Parental Stress Provoked by Short-Term School Closures During the Second COVID-19 Lockdown by Isabelle May, Sarah Awad, Matthias S. May and Albert Ziegler in Journal of Family Issues
