# Supplementary material for: Parental Stress Provoked by Short-Term School Closures During the Second COVID-19 Lockdown
Source: J Fam Issues. 2023 Jan;44(1):25–45. doi: 10.1177/0192513X211041987 (PMC9760518; doi:10.1177/0192513X211041987)
Supplement: sj-pdf-1-jfi-10.1177_0192513X211041987 – Supplemental Material for Parental Stress Provoked by Short-Term School Closures During the Second COVID-19 Lockdown [file sj-pdf-1-jfi-10.1177_0192513X211041987.pdf]

1.Working time

Full - Part-time 50%≤ - Part-time ＞ 50% - not in employment

2.Place of work

100% Homeoffice - Switching between HO and presence - 100% presence (outside)

3. How many children do you have?

4. Child and Class:

5. How many hours do you use to support distance learning on a day of instruction?

6. It's just a problem for me to spend the time to support my children's learning

Does not apply - It is not true - Is truer - Applies fully

7. Information on the child age class

8. My child communicates with the teacher

a. Via mail	Never	Rarely	Occasionally	Regularly
b. Via video chat	Never	Rarely	Occasionally	Regularly
c. Via phone	Never	Rarely	Occasionally	Regularly

9. I communicate with the teacher

a. Via mail	Never	Rarely	Occasionally	Regularly
b. Via video chat	Never	Rarely	Occasionally	Regularly
c. Via phone	Never	Rarely	Occasionally	Regularly

10. The teacher corrects the tasks from the homeschooling.

Never Rarely Occasionally Regularly

11.My child got a plan before homeschooling (days/weekly schedule)

Never Rarely Occasionally Regularly

12. My child lacks personal exchange with classmates and teachers

Does not apply It is not true Is truer Applies fully

13. My child's teachers are easily accessible during school closure

Does not apply It is not true Is truer Applies fully

14. I know who I can contact to get help if I have problems assisting my child.

Does not apply It is not true Is truer Applies fully

15. When learning at home, I support my child in

a. Motivation ("Please start now, keep going ")	Regularly	Occasionally	Rarely	Never
b. Scheduling ("First you work maths, then...")	Regularly	Occasionally	Rarely	Never
c. Technical problems	Regularly	Occasionally	Rarely	Never
d. Explanation	Regularly	Occasionally	Rarely	Never

16. Others support my child in case of emerging problems in the following areas (ET1 ET2, grandparents, siblings, others)

a. Motivation	Regularly	Occasionally	Rarely	Never
b.Structure of the lesson day	Regularly	Occasionally	Rarely	Never
c.Technical problems	Regularly	Occasionally	Rarely	Never

17. My child and I often argue about learning during school closures

Does not apply at all It's not true Is truer Applies fully

18a. My child liked to go to school

Does not apply at all It's not true Is truer Applies fully

b. My child did its homework, in typical school operation, quickly and alone

Does not apply at all It's not true Is truer Applies fully

c. My child and I often argued about learning

Does not apply at all It's not true Is truer Applies fully

d. My child could self-organize his learning well

Doesn't apply at all It's not true Is truer Applies fully

19.Before homeschooling (during the classroom) I supported my child in the following areas

a. Motivation	Regularly	Occasionally	Rarely	Never
b. Scheduling	Regularly	Occasionally	Rarely	Never
c.Technical problems	Regularly	Occasionally	Rarely	Never
d.Explanation	Regularly	Occasionally	Rarely	Never

20.I feel stressed and overwhelmed in the following areas in supporting my child

a. Motivation	Doesn't apply	It is not true	Is truer	Applies fully
b. scheduling	Doesn't apply	It is not true	Is truer	Applies fully
c. Technical problems	Doesn't apply	It is not true	Is truer	Applies fully
d. Explanation	Doesn't apply	It is not true	Is truer	Applies fully

21. I am concerned that my child will be closed again by the closure of the school ...

a. Knowledge gaps	Doesn't apply	It is not true	Is truer	Applies fully
b. lack of social skills	Doesn't apply	It is not true	Is truer	Applies fully
c. motivation loses	Doesn't apply	It is not true	Is truer	Applies fully

22a. I often feel left alone when I support homeschooling

Does not apply It is not true Is truer Applies fully

b. Being a mother/father is harder than it was before the pandemic.

Does not apply It is not true Is truer Applies fully

c. I feel constrained by the additional responsibility for learning at home.

Does not apply It is not true Is truer Applies fully

23.I would like to receive the following help from the school:

Reduction of the volume of material for the whole school year	Yes	No
Reduction of proof of performance	Yes	No
More time to repeat and consolidate	Yes	No
Uniform technical design	Yes	No
Shared virtual start and end of the day	Yes	No
Virtual Parents' Evening	Yes	No
Timely feedback (corrections of tasks)	Yes	No
Individual feedback (corrections of tasks)	Yes	No

24.It would help me if

a mandatory start of the day every day (e.g., 8 a.m. Videoconference)	Yes	No
a single, binding videoconferencing program	Yes	No
the school would provide the hardware	Yes	No
regular feedback from the teacher on how to handle my child's tasks	Yes	No
uniform structuring aids for the children (day/weekly schedule with time)	Yes	No
binding deadlines for tasks to be communicated to the children	Yes	No

25. My child can learn just as well at home as in face-to-face classes.

Doesn't apply at all It's not true Is truer Applies fully

26. State

27.Gender M W D

28.Age 18 - 30 - 40 41 – 50 50

29.Educational attainment No professional qualification

30.Family situation Single parent Divorced alternating model 2Parent family

31. Relationship with child Mother, Father, Step- Relationship, Grandparents, Others: _
